# Ouroboros resembling competitive endogenous loop (ORCEL) in circular RNAs revealed through transcriptome sequencing dataset analysis

**DOI:** 10.1186/s12864-018-4456-9

**Published:** 2018-05-09

**Authors:** Yu-Chen Liu, Hsiao-Chin Hong, Chi-Dung Yang, Wei-Hsiang Lee, Hsin-Tzu Huang, Hsien-Da Huang

**Affiliations:** 1 Institute of Engineering in Medicine, University of California, La Jolla, San Diego, CA USA; 2 Institute of Bioinformatics and Systems Biology, National Chiao Tung University, HsinChu, Taiwan; 3 Institute of Pharmacology, National Yang-Ming University, Taipei, Taiwan; 4 Institute of Population Health Sciences, National Health Research Institutes, Miaoli, Taiwan; 5 Department of Biological Science and Technology, Institute of Bioinformatics, National Chiao Tung University, Hsin-Chu, Taiwan; 6 Department of Medical Research, Chung Shan Medical University Hospital, Taichung, Taiwan

**Keywords:** CircRNA, CircNet, CERNA, miRNA sponge, ORCEL

## Abstract

**Background:**

Emerging evidence indicates that Circular RNAs (circRNAs) exert post-transcriptional regulation of gene expression. A subclass of circRNA was found enriched with miRNA target sites. This evidence suggests that this kind of circRNA functions as natural miRNA sponge. Noticing the potential impacts of circular RNA research, we were motivated to identify novel circRNAs as well as putative circRNA-miRNA interactions through retroactive sourced transcriptome sequencing samples.

**Results:**

Through the analysis in 465 RNA-seq runs and 22 reports published in recent years, putatively circRNA sponged miRNA that had been experimentally verified targeting circRNA host gene were found. From this observation, supporting evidence of the competitive endogenous relationship of circRNAs and miRNAs targeting circRNA host genes can be observed. Given the self-regulation and self-induction nature of these circRNAs, this kind of hypothetical phenomenon was hereby called Ouroboros Resembling Competitive Endogenous Loop (ORCEL) in circular RNAs.

**Conclusions:**

The fact that miRNA sponge circRNA originated from region miRNA target sites enriched regions, while genes encoded from these regions are conserved to be miRNA targets rationalize the existence of ORCEL.

**Electronic supplementary material:**

The online version of this article (10.1186/s12864-018-4456-9) contains supplementary material, which is available to authorized users.

## Background

More than 50 years have passed since H. Harris deduced that most nuclear RNAs are likely to be non-protein-coding in 1959 [1]. Existence of functional noncoding RNAs has become convention knowledge. Thanks to the dramatically expanded scope of transcriptomics research with high throughput sequencing technology developed in recent years [2], it is possible now to more accurately investigate the expression of non-coding RNA. Differential expressed long non coding RNAs have been found and reported in almost a weekly basis [3]. Among this trend of digging into retroactive sourced experimental data, circular RNAs emerge on stump eventually.

Circular RNAs (circRNAs) represent a type of regulatory noncoding RNA whose head 3′ and tail 5′ ends covalently bond together to result in a circular form. The circular form was verified with electron microscope in 1979 [4]. In 2012, Salzman et al. [5, 6] developed an algorithm to detect scrambled exons in RNA-Seq datasets, and reported that circular RNA isoforms are actually predominant in many human gene isoforms. Later, an improved version of the algorithm with exon splicing site AU/AC searching was applied to find the fact that circRNAs serving as natural microRNA “sponges”, which enriched with miRNA targeting site and serving competitive endogenous RNAs (ceRNAs) [7, 8]. A circRNA named CDR1as [9] expressed in human and mouse brain was shown to negatively regulate miR-7 in a post-transcriptional manner [7, 9, 10]; this mechanism appears to be evolutionarily conserved [7]. With the regulation potential of miRNAs, circRNAs became widely interested in the research field [11]. Circular RNA can be enriched within the sample through treating samples with RNase R before conducting RNA-Seq [12, 13].

In the following years, extended identification of circRNAs in mouse [14], fly [15] and other animals [16] suggests that circRNA ubiquity is evolutionally-conserved. The reported circRNAs are not results of singular case. These experiment results tend to be reproducible. Further evidence indicates that human circRNA expression exhibits tissue specificity, and now tens of thousands of circRNAs have been found and reported across human tissues [14, 17–22].

Aware of the significance of circRNAs research, in 2015, we constructed a database called CircNet [23] to not only collect the published data, but also extend the scope of reported circRNAs and provide resources to aid in field.

To acquire an enriched collection of human circular RNAs, previously reported and newly identified human circRNAs were collected in this study. Reported human back-spliced junction sites were also collected from 22 recent studies [6, 7, 13, 20, 22, 24–39]. In addition, 465 transcriptome sequencing data sets were collected from NCBI Sequence Read Archive [23, 40], including datasets used in recent publications [14, 18–22, 38].

Circular RNA back-spliced junction sites were identified within these samples. To acquire the expression patterns of circRNAs within these samples, a pipeline was developed to annotate the sequence of the circRNAs. With the putative sequences of circRNAs, miRNA target prediction was conducted on these sequences. Transcripts abundance within the samples was conducted through the transcript deconvolution algorithm [41].

Through analyzing the collect circRNA, combined with the annotation and miRNA sponge prediction, we found that in 728 human circRNA host genes, the generated circRNAs were found to have high affinity to sponge miRNAs which were reported to target these genes. The calculated *P*-value of the circRNA and miRNA pairs are lower than 0.005. From this observation, existence of competitive endogenous loop of circRNAs and their host gene can be observed. Given the self-regulation and self-induction nature of these circRNAs, it was hereby named Ouroboros Resembling Competitive Endogenous Loop (ORCEL) in circular RNAs.

## Results

Through the expression profiling of circRNA, combined with the previously reported back spliced junction sites, we found 2747 circRNAs originated from 2693 back spliced junction sites that meet the defined threshold. From the analysis, we found that in 728 human circRNA host genes, including 909 circRNA transcripts, the generated circRNAs were found to have high affinity to sponge miRNAs. Meanwhile these miRNAs were reported to target the circRNA originated genes.

### Annotation of circRNA sequence

From our analysis, we found 2693 back splice junction sites that meet the defined threshold on human genome. The combined amount of peer review reports and RNA-Seq samples in which these junction sites were found or reported ranges from 10 to 126. The miRNA sponge CDR1as [9] was found in over 100 of our collected samples. A detail list of these circRNAs, as well as their corresponding SRPBM and FPKM values within the samples can be found in Additional file 1. The data was also updated into our public database CircNet [23] in late 2016.

### Self-regulation and self-induction nature of ORCEL

With the observation of most circRNAs originated from circularization of coding gene exons, it was deducted that circRNA biogenesis competes with pre-mRNA splicing [14]. As an isoform originated from part of gene locus, expression of circRNAs correlates with their originated genes. While on the other hand, the post-transcriptional roles of miRNAs to most of the genes has become a conventional knowledge [42]. Result of our analysis suggest the existence of competitive endogenous loop of circRNAs and their host genes. In the sequences of 909 circRNA transcripts from 728 human genes, we found enriched miRNA targeting sites. Echo to the recent evidences suggesting that one circRNA can sponge multiple different miRNAs [34], we found 1112 miRNAs that can be sponged by these circRNAs. Meanwhile these miRNAs were also found to target the circRNA originated genes. Hence a looping regulative relationship among circRNA, circRNA originated gene and miRNA targeting the gene was found. Given the self-regulation and self-induction nature of these circRNAs, the phenomenon was hereby named Ouroboros Resembling Competitive Endogenous Loop (ORCEL) in circular RNAs. The term was inspired by Friedrich August Kekulé and his famous discovery of benzene ring. A complete list of ORCEL is available in Additional file 2.

### Enrichment analysis of the ORCEL genes

**Fig. 1:**
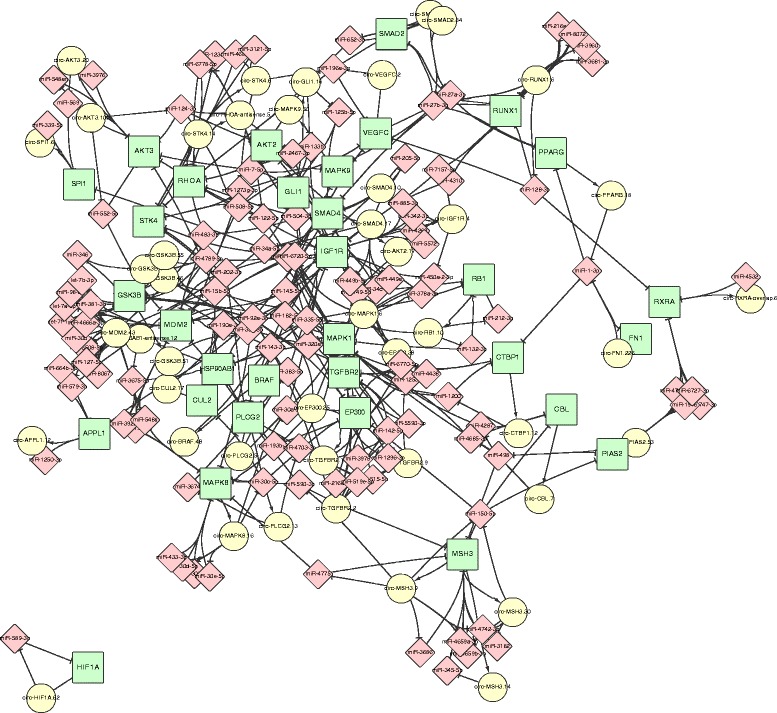
Network of 32 ORCEL genes enriched in pathways in cancer. The genes are illustrated as green rectangle nodes. The circRNAs are illustrated as yellow circles. The miRNAs involved are illustrated as pink diamond nodes

An enrichment analysis of the 728 ORCEL genes was conducted through DAVID [43]. In the result of this analysis we found that many ORCEL genes participate in important KEGG [44] pathways such as Ubiquitin mediated proteolysis, Pathways in cancer, Focal adhesion and Progesterone-mediated oocyte maturation, as summarized in Table 1. In the 32 genes participated in the hsa05200: Pathways in cancer, 115 miRNAs and 45 circRNA transcripts were found participated in the ORCEL, as illustrated in the network of Fig. 1. The network was generated through Cytoscape [45].

**Table 1:** KEGG pathway enrichment of ORCEL genes

KEGG Pathway	No. of Genes	*P*-value
hsa04120:Ubiquitin mediated proteolysis	19	2.14E-05
hsa05200:Pathways in cancer	32	2.59E-05
hsa04510:Focal adhesion	23	4.83E-05
hsa04914:Progesterone-mediated oocyte maturation	13	2.97E-04

The ORCEL genes enriched in KEGG pathways with significant *P* values

**Fig. 2:**
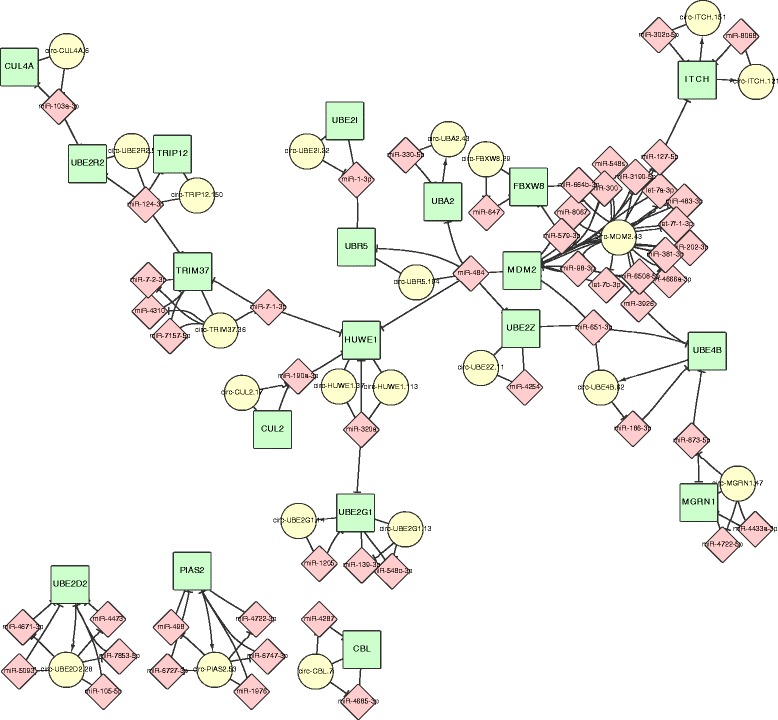
Network of 19 ORCEL genes enriched in ubiquitin mediated proteolysis. The genes are illustrated as green rectangle nodes. The circRNAs are illustrated as yellow circles. The miRNAs involved are illustrated as pink diamond nodes

From the network illustrated in Fig. 1, it can be noticed that among the 32 genes enriched in the pathways in cancer, only the ORCEL of HIA1 doesn’t involve miRNAs targeting multiple other genes involved in the pathway. A complete table of the enrichment can be found in Additional file 3. In the network illustrated in Fig. 2, for the 19 genes participated in the hsa04120: Ubiquitin mediated proteolysis, 52 miRNAs and 22 circRNA transcripts were found participated in the ORCEL. It is also worth mentioned that 61 ORCEL genes were enriched in the GO term GO:0046907~intracellular transport and 121 ORCEL genes were enriched in GO:0031974~membrane-enclosed lumen. The ORCEL phenomenon can potential be correlated to intracellular transport.

## Discussion

In this study, putative roles of circRNAs serving as endogenous miRNA sponges were investigated through analysis of transcriptome sequencing data sets and published results. With the head-tail junction structure, circular RNAs are more stable than other kind of long noncoding RNAs. Hence circRNAs are hypothetically easier to acuminate in cells. The longer half-lives of circRNAs allow the presence of miRNA target sites increase within the cells. Through statistical analysis of the abundance of the miRNA target seeds on circRNA sequences, putative circRNA serving as miRNA sponges were identified. Recent years experiment results suggest certain threshold of miRNA target sites needs to be reached for the ceRNAs to have physiological effects [46, 47], henceforth the analysis was focused on the abundance of miRNA target sites. The application of FPKM of the putative circRNA sequences, and SRPBM of the back spliced junction sites as thresholds should increase the prudence of the circRNA sequence prediction in this study. With the further compliance with results of recent year studies, results of our analysis suggest that only a certain subset of expressed circRNAs potentially serve as nature miRNA sponges. Among the miRNAs predicted to be sponged by these identified circRNAs, many had been experimentally verified to target the circRNA source genes. From these observations we hypothesize that genes targeted by miRNAs tend to be conserved with enriched miRNA targeting site in the coding region. CircRNAs coded from these regions can henceforth sponge the miRNAs when overexpressed. This phenomenon was hereby named Ouroboros Resembling Competitive Endogenous Loop (ORCEL) in circular RNAs. The term was inspired by Friedrich August Kekulé and his famous discovery of benzene ring [48]. Given the observation of this phenomena in genes involved in cancer pathways, validation of this hypothetical phenomena shall significantly impact the research prospects in medical science. ORCEL can potentially serving as a kind of control mechanism to resist miRNAs overdose. The fact that miRNA sponge circRNA originated from region miRNA target sites enriched regions, while genes encoded from these regions are conserved to be miRNA targets rationalize the existence of ORCEL.

## Conclusions

Through the bioinformatics analysis it was found that for certain subset of circRNAs, putatively sponged miRNA had been experimentally verified targeting circRNA host gene. From this observation, the existence of competitive endogenous loop of circRNAs and their host gene can be observed. Given the self-regulation and self-induction nature of these circRNAs, this kind of phenomenon was hereby called Ouroboros Resembling Competitive Endogenous Loop (ORCEL) in circular RNAs.

## Methods

**Fig. 3:**
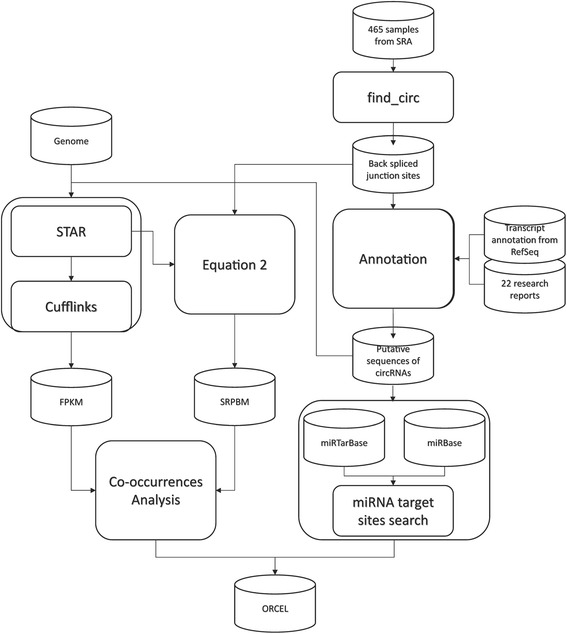
Overview of the data analysis process in this research. The general view of the process of ORCEL discovery is illustrated in this figure

The data analysis process of this research is summarized in Fig. 3. First, to identify circRNA, transcriptome sequencing data sets were obtained from the NCBI Sequence Read Archive (SRA). The back-spliced junction sites in each RNA-seq sample were identified using a circRNA discovery pipeline adapting the scripts provided on circBase [7, 49], which was referred as find_circ [50]. Detected back-spliced junction sites, along with the collected junction sites from previous reports, were further compared with the hg19 human genome annotation from RefSeq to annotate circRNA isoform sequence. The annotated sequence were then applied in the prediction of circRNA-miRNA interactions. Occurrence of miRNA target seeds in circRNA isoforms were examined and normalized by isoform length. The significance of interactions was evaluated by referring to the background distribution of miRNA seeds in all transcripts and only circRNA-miRNA interactions with *P*-values < 0.005 were collected. Expression profiling of the circRNAs within each of the samples collected from SRA was conducted in two different approaches: normalized counts of reads spanning the back spliced junction sites SRPBM [13] and normalized counts of reads aligned on the annotated sequences of circRNAs in units of FPKM. Only the circRNAs with estimated expression level over the threshold and found in multiple researches or samples were analyzed in this study.

### Detection of the back spliced junction sites

Reported human back-spliced junction sites were collected from 22 recent studies [6, 7, 13, 20, 22, 24–39]. In addition, 465 transcriptome sequencing data sets were collected from NCBI Sequence Read Archive [23, 40]. The back-spliced junction sites in each RNA-seq sample were identified using a circRNA discovery pipeline referred as find_circ [7, 49, 50]. We apply the criteria defined in the pipeline hence the detected junction sites met same standards as those in the previous reports, as described in the Memczak et al. 2013 study.

### Annotation of circRNA full sequence

The method was described in our previous study [23]. The method was further applied on the updated data from reports between year 2014 and 2016. To acquire the full length nucleotide sequence from RNA-seq reads, back-spliced junction sites were compared with the hg19 human genome annotation as obtained from UCSC genome browser and RefSeq [51, 52]. Given the results of recent research, multiple circRNA isoforms might originate from the same back-spliced junction site [6, 53]. Hence we annotated multiple circRNA isoforms for one back-spliced junction site. The annotation was conducted following the guideline:

1. For the back spliced junction sites locate on exact “head” and “tail” locus of exons from same transcript from RefSeq [51, 52], all the flanking exons of the transcript were considered as part of the same circRNA.

2 $$ SRPBM=\frac{Read s\ count\times {10}^9}{Read\ lenth\times Mapped\ reads} $$

(2) For those back spliced junction sites flanked multiple isoforms from RefSeq [51, 52], existence of multiple isoform of circRNA was assumed.
(3) For those circRNAs associated with these back-spliced junction sites having small misalignments to exon locations, flanked exons and a small portion of intron sequence locating in the head or tail locations were considered as parts of the isoforms.
(4) For the junction sites that were found to be located in intergenic positions while others, despite overlapping with certain genes, localized to their antisense strands, the entire flanked sequence was considered as the sequence of the circRNA.

The resulted sequence annotation was took for expression analysis and miRNA target search. The annotation along with the gene transcripts was recorded in gtf format for the expression profiling.

### Identification of potential miRNA sponges

Developed from results of our previous study [23, 54], to find the potential miRNA and circRNA interactions, we conducted a statistical analysis on the amount of miRNA binding sites on the annotated circRNAs sequences.

The miRNA target sequences deemed typical: 6mer, 7mer-A1, 7mer-m8 and 8mer sequences [42] were extracted from miRBase [55]. Perfect complementarity sites were found on the annotated circRNA as well as gene transcripts sequences through iterative searching. To normalize the number of occurrences of these sites by the length of the transcripts, the following formula was used:

1 $$ \mathrm{Frequency}\ \mathrm{of}\ \mathrm{Nmer}=\frac{Number\ of\ target\ seeds\times 1000}{N\times Length\ of\ CircRNA} $$

Where the ‘N’ is the length of the seed. *N* = 6 for 6mer, *N* = 7 for 7mers and 8 for 8mer. With this formula, four frequency numbers can be acquired from each pair of circRNA and miRNA. To distinguish circRNA from linear isoforms, frequency values were also calculated for gene transcripts mRNA. We calculated all the frequency value of the circRNAs as well as the linear isoforms pairing to miRNAs, and then converted the Z-score of the normal distribution into one tail *P*-value through survival function.

The circRNA-miRNA pair with *P*-value < 0.005 was considered high regulatory potential between the circRNA and miRNA. The miRNAs and experimentally verified gene targets were collected from miRTarBase [54].

### Expression profiling of circRNAs

As previously described, the abundance of the circRNA within the collected samples were estimated through the transcript deconvolution algorithm of the Cufflinks pipeline [41]. To further increase the prudence of circRNA detection within the transcriptome, the normalized counts of sequence reads spanning the back spliced junctions were considered.

### The normalized count of reads on back spliced junctions

To normalize the amount of the normalized sequence reads spanning the junction sites, a concept of spliced reads per billion mapping (SRPBM) was applied [13]. Amount of reads mapped onto hg19 human genome was acquired through the tool STAR [56]. The equation applied to calculate SRPBM is as illustrated in Eq. 2. The junction sites with the value of SRPBM larger than 1.0 were selected.

### Transcript deconvolution of circRNAs

RNA-seq aligner STAR [56] was applied to realign the sequence reads from the 465 RNA-seq samples on human genome. With the forth-mentioned gtf file containing annotated exon locus of circRNAs and mRNAs, and the bam files generated from STAR, we estimated the abundance within the sample of the annotated sequence through Cufflinks [41]. The resulted transcripts with FPKM over 1.0 were selected.

### Co-occurrence analysis of circRNA

From the result of recent year comparison study [50], we deducted that inconsistency between different circRNA detection tools and false discovery of highly abundant circRNA could occur in the result of our analysis. Hence in addition to the two values of estimated abundance of circRNA, we further applied the following conditions:

The amount of previous peer review reports in which the back spliced junction sites were reported.

The amount of samples among the 465 collected samples in which the back spliced junction sites were found meeting the criteria defined in find_circ [7, 49, 50].

Only the circRNAs with the combined amount of these two values over 10 were considered in the analysis of this report.

## Additional files

Additional file 1: A detail list of these circRNAs, as well as their corresponding SRPBM and FPKM values within the samples. (XLSX 528 kb)

Additional file 2: A complete list of ORCEL (Ouroboros Resembling Competitive Endogenous Loop) reported in this article. (XLSX 59 kb)

Additional file 3: A complete table of the enrichment of ORCEL genes. (XLSX 90 kb)

## Abbreviations

CeRNA: Competing endogenous RNAs
circRNA: Circular RNA
FPKM: Fragments Per Kilobase of transcript per Million mapped reads
ORCEL: Ouroboros resembling competitive endogenous loop
SRPBM: Spliced reads per billion mapping

## References

[1] Harris H (1959). Turnover of nuclear and cytoplasmic ribonucleic acid in two types of animal cell, with some further observations on the nucleolus. Biochem J.

[2] Wang Z, Gerstein M, Snyder M (2009). RNA-Seq: a revolutionary tool for transcriptomics. Nat Rev Genet.

[3] Kung JT, Colognori D, Lee JT (2013). Long noncoding RNAs: past, present, and future. Genetics.

[4] Hsu M-T, Coca-Prados M (1979). Electron microscopic evidence for the circular form of RNA in the cytoplasm of eukaryotic cells.

[5] Salzman J, Gawad C, Wang PL, Lacayo N, Brown PO (2012). Circular RNAs are the predominant transcript isoform from hundreds of human genes in diverse cell types. PLoS One.

[6] Salzman J, Chen RE, Olsen MN, Wang PL, Brown PO (2013). Cell-type specific features of circular RNA expression. PLoS Genet.

[7] Memczak S, Jens M, Elefsinioti A, Torti F, Krueger J, Rybak A, Maier L, Mackowiak SD, Gregersen LH, Munschauer M (2013). Circular RNAs are a large class of animal RNAs with regulatory potency. Nature.

[8] Tay Y, Rinn J, Pandolfi PP (2014). The multilayered complexity of ceRNA crosstalk and competition. Nature.

[9] Hansen TB, Wiklund ED, Bramsen JB, Villadsen SB, Statham AL, Clark SJ, Kjems J (2011). miRNA-dependent gene silencing involving Ago2-mediated cleavage of a circular antisense RNA. EMBO J.

[10] Hansen TB, Jensen TI, Clausen BH, Bramsen JB, Finsen B, Damgaard CK, Kjems J (2013). Natural RNA circles function as efficient microRNA sponges. Nature.

[11] Wilusz JE, Sharp PA (2013). A circuitous route to noncoding RNA. Science.

[12] Suzuki H, Zuo Y, Wang J, Zhang MQ, Malhotra A, Mayeda A (2006). Characterization of RNase R-digested cellular RNA source that consists of lariat and circular RNAs from pre-mRNA splicing. Nucleic Acids Res.

[13] Jeck WR, Sorrentino JA, Wang K, Slevin MK, Burd CE, Liu J, Marzluff WF, Sharpless NE (2013). Circular RNAs are abundant, conserved, and associated with ALU repeats. RNA.

[14] Ashwal-Fluss R, Meyer M, Pamudurti NR, Ivanov A, Bartok O, Hanan M, Evantal N, Memczak S, Rajewsky N, Kadener S (2014). circRNA biogenesis competes with pre-mRNA splicing. Mol Cell.

[15] Westholm JO, Miura P, Olson S, Shenker S, Joseph B, Sanfilippo P, Celniker SE, Graveley BR, Lai EC (2014). Genome-wide analysis of drosophila circular RNAs reveals their structural and sequence properties and age-dependent neural accumulation. Cell Rep.

[16] Wang PL, Bao Y, Yee M-C, Barrett SP, Hogan GJ, Olsen MN, Dinneny JR, Brown PO, Salzman J: Circular RNA is expressed across the eukaryotic tree of life. PLoS One 2014, 9(3):e90859. 10.1371/journal.pone.0090859 PMC3946582 24609083

[17] Gao Y, Wang J, Zhao F (2015). CIRI: an efficient and unbiased algorithm for de novo circular RNA identification. Genome Biol.

[18] Tian M, Chen R, Li T, Xiao B. Reduced expression of circRNA hsa_circ_0003159 in gastric cancer and its clinical significance. J Clin Lab Anal. 2017. 10.1002/jcla.22281 PMC6817154 28618205

[19] Bachmayr-Heyda A, Reiner AT, Auer K, Sukhbaatar N, Aust S, Bachleitner-Hofmann T, Mesteri I, Grunt TW, Zeillinger R, Pils D (2015). Correlation of circular RNA abundance with proliferation--exemplified with colorectal and ovarian cancer, idiopathic lung fibrosis, and normal human tissues. Sci Rep-Uk.

[20] Zhang X-O, Wang H-B, Zhang Y, Lu X, Chen L-L, Yang L (2014). Complementary sequence-mediated exon circularization. Cell.

[21] Guo JU, Agarwal V, Guo H, Bartel DP (2014). Expanded identification and characterization of mammalian circular RNAs. Genome Biol.

[22] Caiment F, Gaj S, Claessen S, Kleinjans J (2015). High-throughput data integration of RNA–miRNA–circRNA reveals novel insights into mechanisms of benzo [a] pyrene-induced carcinogenicity. Nucleic Acids Res.

[23] Liu Y-C, Li J-R, Sun C-H, Andrews E, Chao R-F, Lin F-M, Weng S-L, Hsu S-D, Huang C-C, Cheng C (2016). CircNet: a database of circular RNAs derived from transcriptome sequencing data. Nucleic Acids Res.

[24] Boeckel J-N, Jaé N, Heumüller AW, Chen W, Boon RA, Stellos K, Zeiher AM, John D, Uchida S, Dimmeler S (2015). Identification and characterization of hypoxia-regulated endothelial circular RNA. Circ Res.

[25] Bahn JH, Zhang Q, Li F, Chan T-M, Lin X, Kim Y, Wong DT, Xiao X (2015). The landscape of microRNA, Piwi-interacting RNA, and circular RNA in human saliva. Clin Chem.

[26] Bachmayr-Heyda A, Reiner AT, Auer K, Sukhbaatar N, Aust S, Bachleitner-Hofmann T, Mesteri I, Grunt TW, Zeillinger R, Pils D (2015). Correlation of circular RNA abundance with proliferation-exemplified with colorectal and ovarian cancer, idiopathic lung fibrosis, and normal human tissues. Scientific reports..

[27] Conn SJ, Pillman KA, Toubia J, Conn VM, Salmanidis M, Phillips CA, Roslan S, Schreiber AW, Gregory PA, Goodall GJ (2015). The RNA binding protein quaking regulates formation of circRNAs. Cell.

[28] Memczak S, Papavasileiou P, Peters O, Rajewsky N (2015). Identification and characterization of circular RNAs as a new class of putative biomarkers in human blood. PLoS One.

[29] Alhasan AA, Izuogu OG, Al-Balool HH, Steyn JS, Evans A, Colzani M, Ghevaert C, Mountford JC, Marenah L, Elliott DJ (2016). Circular RNA enrichment in platelets is a signature of transcriptome degradation. Blood.

[30] Cheng J, Metge F, Dieterich C (2016). Specific identification and quantification of circular RNAs from sequencing data. Bioinformatics.

[31] Zhang XO, Dong R, Zhang Y, Zhang JL, Luo Z, Zhang J, Chen LL, Yang L (2016). Diverse alternative back-splicing and alternative splicing landscape of circular RNAs. Genome Res.

[32] Song X, Zhang N, Han P, Moon BS, Lai RK, Wang K, Lu W (2016). Circular RNA profile in gliomas revealed by identification tool UROBORUS. Nucleic Acids Res.

[33] Dang Y, Yan L, Hu B, Fan X, Ren Y, Li R, Lian Y, Yan J, Li Q, Zhang Y (2016). Tracing the expression of circular RNAs in human pre-implantation embryos. Genome Biol.

[34] Zheng Q, Bao C, Guo W, Li S, Chen J, Chen B, Luo Y, Lyu D, Li Y, Shi G. Circular RNA profiling reveals an abundant circHIPK3 that regulates cell growth by sponging multiple miRNAs. Nature communications. 2016;7. 10.1038/ncomms11215 PMC4823868 27050392

[35] Zhang Y, Zhang X-O, Chen T, Xiang J-F, Yin Q-F, Xing Y-H, Zhu S, Yang L, Chen L-L (2013). Circular intronic long noncoding RNAs. Mol Cell.

[36] Rybak-Wolf A, Stottmeister C, Glažar P, Jens M, Pino N, Giusti S, Hanan M, Behm M, Bartok O, Ashwal-Fluss R (2015). Circular RNAs in the mammalian brain are highly abundant, conserved, and dynamically expressed. Mol Cell.

[37] Guo JU, Agarwal V, Guo H, Bartel DP (2014). Expanded identification and characterization of mammalian circular RNAs. Genome Biol.

[38] Gao Y, Wang J, Zhao F (2015). CIRI: an efficient and unbiased algorithm for de novo circular RNA identification. Genome Biol.

[39] Kelly S, Greenman C, Cook PR, Papantonis A (2015). Exon skipping is correlated with exon circularization. J Mol Biol.

[40] Leinonen R, Sugawara H, Shumway M (2011). International Nucleotide Sequence Database C. The sequence read archive. Nucleic Acids Res.

[41] Trapnell C, Roberts A, Goff L, Pertea G, Kim D, Kelley DR, Pimentel H, Salzberg SL, Rinn JL, Pachter L (2012). Differential gene and transcript expression analysis of RNA-seq experiments with TopHat and cufflinks. Nat Protoc.

[42] Bartel DP (2009). MicroRNAs: target recognition and regulatory functions. Cell.

[43] Dennis G, Sherman BT, Hosack DA, Yang J, Gao W, Lane HC, Lempicki RA (2003). DAVID: database for annotation, visualization, and integrated discovery. Genome Biol.

[44] Kanehisa M, Goto S (2000). KEGG: kyoto encyclopedia of genes and genomes. Nucleic Acids Res.

[45] Shannon P, Markiel A, Ozier O, Baliga NS, Wang JT, Ramage D, Amin N, Schwikowski B, Ideker T (2003). Cytoscape: a software environment for integrated models of biomolecular interaction networks. Genome Res.

[46] Denzler R, Agarwal V, Stefano J, Bartel DP, Stoffel M (2014). Assessing the ceRNA hypothesis with quantitative measurements of miRNA and target abundance. Mol Cell.

[47] Thomson DW, Dinger ME (2016). Endogenous microRNA sponges: evidence and controversy. Nat Rev Genet.

[48] Brock WH (1996). August Kekulé (1829–1896): theoretical chemist. Endeavour.

[49] Glažar P, Papavasileiou P, Rajewsky N (2014). circBase: a database for circular RNAs. RNA.

[50] Hansen TB, Venø MT, Damgaard CK, Kjems J (2016). Comparison of circular RNA prediction tools. Nucleic Acids Res.

[51] Karolchik D, Baertsch R, Diekhans M, Furey TS, Hinrichs A, Lu Y, Roskin KM, Schwartz M, Sugnet CW, Thomas DJ (2003). The UCSC genome browser database. Nucleic Acids Res.

[52] Pruitt KD, Tatusova T, Maglott DR (2007). NCBI reference sequences (RefSeq): a curated non-redundant sequence database of genomes, transcripts and proteins. Nucleic Acids Res.

[53] You X, Vlatkovic I, Babic A, Will T, Epstein I, Tushev G, Akbalik G, Wang M, Glock C, Quedenau C (2015). Neural circular RNAs are derived from synaptic genes and regulated by development and plasticity. Nat Neurosci.

[54] Chou C-H, Chang N-W, Shrestha S, Hsu S-D, Lin Y-L, Lee W-H, Yang C-D, Hong H-C, Wei T-Y, Tu S-J (2016). miRTarBase 2016: updates to the experimentally validated miRNA-target interactions database. Nucleic Acids Res.

[55] Griffiths-Jones S, Grocock RJ, Van Dongen S, Bateman A, Enright AJ (2006). miRBase: microRNA sequences, targets and gene nomenclature. Nucleic Acids Res.

[56] Dobin A, Davis CA, Schlesinger F, Drenkow J, Zaleski C, Jha S, Batut P, Chaisson M, Gingeras TR (2013). STAR: ultrafast universal RNA-seq aligner. Bioinformatics.

